# Using ChatGPT to assist in judging the indications for emergency ultrasound: an innovative exploration of optimizing medical resource allocation

**DOI:** 10.3389/fmed.2025.1567608

**Published:** 2025-06-18

**Authors:** Zhirong Xu, Jiayi Ye, Jiemin Chen, Xiaoqian Zhang, Jiawei Wang

**Affiliations:** ^1^Department of Ultrasound, The Second Affiliated Hospital of Fujian Medical University, Quanzhou, China; ^2^Department of Nuclear Medicine, The Second Affiliated Hospital of Fujian Medical University, Quanzhou, China; ^3^Department of Emergency, The Second Affiliated Hospital of Fujian Medical University, Quanzhou, China

**Keywords:** artificial intelligence, emergency ultrasound, GPT-4O, medical resource allocation, AI in healthcare, ultrasound

## Abstract

**Introduction:**

To assess the performance of the GPT-4O model in determining the indications for emergency ultrasound and to explore its potential for optimizing medical resource allocation.

**Methods:**

This single-center retrospective observational study included 200 patients who underwent emergency ultrasound at the emergency department. Senior clinicians assessed the indications for ultrasound based on guidelines, which served as the gold standard. The medical records were input into the GPT-4O model, which generated binary classification results. The model’s performance was analyzed using confusion matrices and ROC curves.

**Results:**

The GPT-4O model achieved perfect sensitivity and NPV (1.00), with specificity and PPV of 0.86, and an AUC of 0.93. The model accurately identified 92 emergency cases and 93 non-emergency cases, with only 15 non-emergency cases misclassified as emergency cases.

**Conclusion:**

The GPT-4O model showed excellent performance in determining the indications for emergency ultrasound, particularly in terms of sensitivity and negative predictive value. It has the potential to reduce unnecessary examinations and optimize the allocation of medical resources.

## 1 Introduction

Point-of-care ultrasound (POCUS) is a critical diagnostic tool in emergency medicine, known for its real-time, non-invasive, and efficient nature. It is widely used in the rapid evaluation of high-risk conditions such as acute chest pain, abdominal pain, and trauma (1–4). However, the phenomenon of “false emergencies” – ultrasound exams that do not meet the criteria for urgent use – is common, leading to a waste of medical resources and potentially delaying care for patients in genuine need. The standardization of emergency ultrasound use, the reduction of unnecessary examinations, and the optimization of medical resource allocation have become pressing challenges in the field of emergency medicine (5).

In recent years, artificial intelligence (AI), especially natural language processing (NLP) technology, has shown immense potential in healthcare. Large language models like GPT-4 have supported clinical decision-making by analyzing complex data and understanding clinical contexts. For example, GPT-4 has demonstrated similar decision-making consistency to that of physicians in emergency department scoring systems, such as the NIHSS, HEART score, and Alvarado score (6). Recent studies have also highlighted the utility of multimodal GPT models in ultrasound interpretation and clinical report verification, underscoring the broader diagnostic value of such AI systems in real-world settings (7, 8). According to the ACEP guidelines, the indications for emergency ultrasound are clearly categorized into five main areas: resuscitation, diagnosis, symptom- or sign-based applications, procedural guidance, and monitoring/therapy. These guidelines provide a clear framework for AI-assisted determination of emergency ultrasound indications (9).

This study aims to explore the application of the AI model, GPT-4O, in determining emergency ultrasound indications. The goal is to evaluate its efficacy in standardizing ultrasound use, enhancing diagnostic efficiency, and optimizing medical resource allocation. This research not only provides technological support for rationalizing emergency ultrasound usage but also lays the foundation for integrating AI into medical resource management. This study is the first to evaluate the application of GPT-4O in determining emergency ultrasound indications, offering a novel approach to optimizing medical resource allocation in high-pressure emergency settings.

## 2 Materials and methods

### 2.1 Study design

This single-center retrospective observational study aimed to evaluate the performance of the GPT-4O model in determining emergency ultrasound indications. The study data were collected from 200 patients who visited the emergency department and underwent ultrasound between 6 December and 12 December 2024, at the Fujian Medical University Affiliated Second Hospital.

The inclusion criteria were as follows: (1) patients who underwent emergency ultrasound; (2) complete medical records, including chief complaint, medical history, physical signs, and auxiliary examination results.

The exclusion criteria were as follows: incomplete medical records or missing key data.

The Ethics Committee of the Second Affiliated Hospital of Fujian Medical University approved this study (89–203), and all patients provided written informed consent.

### 2.2 Study procedure

Each case was presented to GPT-4O via the following prompt: “You are a professional emergency physician. Based on the following case, please determine whether this patient meets the indications for emergency ultrasound.”

Each medical record was preprocessed into a structured format to ensure consistency. The input included the following key elements: (1) patient demographics (age, gender), (2) chief complaint, (3) history of present illness, (4) vital signs (e.g., blood pressure, heart rate, temperature), (5) relevant laboratory and imaging findings, and (6) initial diagnosis and planned management.

**FIGURE 1:**
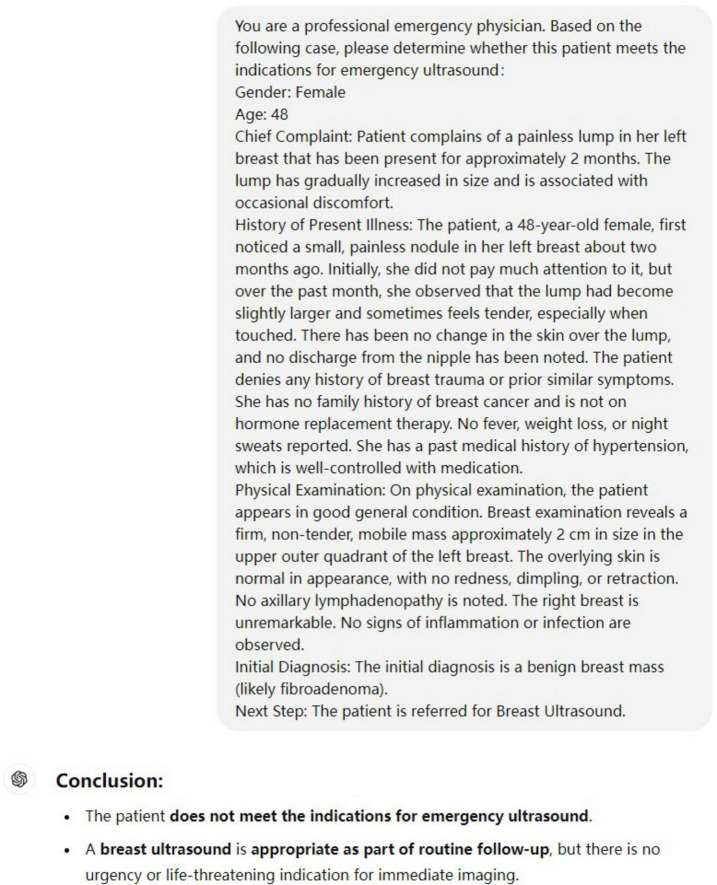
Example of a standardized input case and GPT-4O output. The input includes demographics, chief complaint, history of present illness, physical examination, and initial diagnosis. This format reflects how each case was submitted for model evaluation.

The prompt explicitly instructed the model to assess indications based only on acute symptoms. A representative example of this input format is provided in Figure 1.

### 2.3 Criteria for emergency ultrasound indications

The indications for emergency ultrasound were based on the Ultrasound Guidelines: Emergency, Point-of-Care, and Clinical Ultrasound Guidelines in Medicine. Two experienced clinicians independently assessed whether each case met the criteria. If there was a disagreement, a third physician made the final decision based on the guidelines. The evaluation metrics included confusion matrices, sensitivity, specificity, accuracy, and ROC curve analysis.

### 2.4 Statistical analysis

Statistical analysis was conducted using Python version 3.8.0 (Python Software Foundation, Beaverton, Oregon, United States). The model’s performance was assessed through confusion matrices and ROC curve analysis. Sensitivity, specificity, positive predictive value (PPV), negative predictive value (NPV), and area under curve (AUC) were calculated to comprehensively evaluate the model’s performance in determining emergency ultrasound indications.

## 3 Results

### 3.1 Case characteristics

A total of 200 emergency ultrasound cases were included, of which 92 met the emergency ultrasound indications, and 108 did not.

### 3.2 Confusion matrix

**FIGURE 2:**
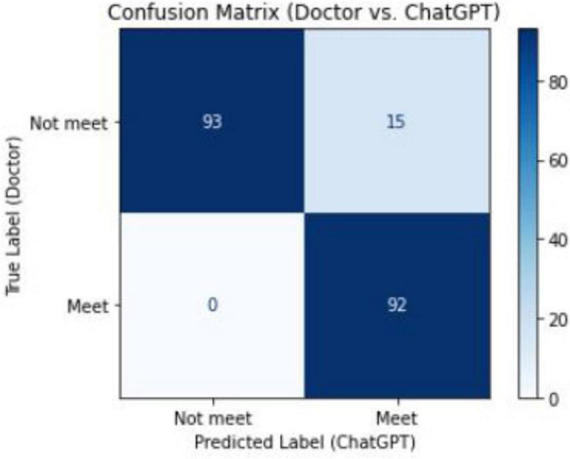
Confusion matrix between senior physicians and ChatGPT in the classification of emergency ultrasound indications.

Compared with the senior clinicians’ judgments, the GPT-4O model accurately identified 92 emergency cases and 93 non-emergency cases. The model misclassified 15 non-emergency cases as emergency cases but did not incorrectly classify any emergency cases as non-emergency (Figure 2).

### 3.3 Misclassification analysis

Among the 200 cases, 15 non-emergency cases were misclassified as emergency by the model. A qualitative review revealed three major patterns of misclassification: (1) Chronic symptom misinterpretation: Several patients with long-standing but stable conditions (e.g., chronic diarrhea or breast masses) were labeled incorrectly due to the model overestimating urgency. (2) Multiple simultaneous ultrasound indications: In some patients, emergency and non-emergency indications coexisted. The model lacked granularity to differentiate between types of scans. (3) Non-specific symptom presentation: Cases with vague descriptions (e.g., “discomfort”) led to ambiguity in classification. These findings highlight areas for prompt refinement and case stratification in future iterations.

### 3.4 ROC curve

**FIGURE 3:**
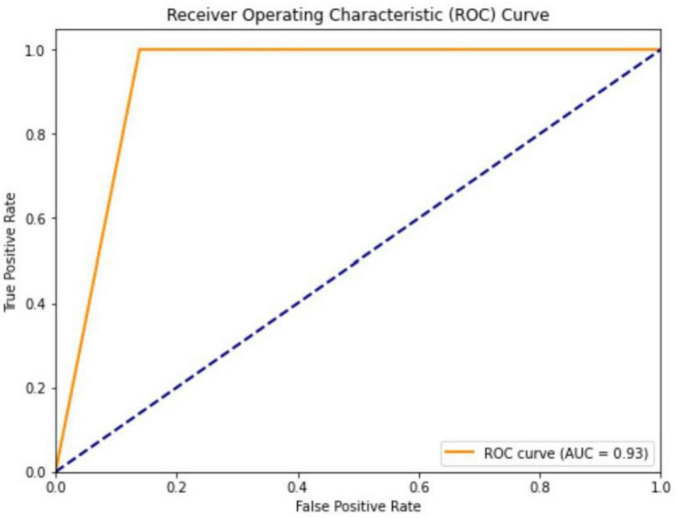
ChatGPT receiver operating characteristic curve [area under curve (AUC) = 0.93].

The ROC analysis revealed an AUC value of 0.93 for the GPT-4O model, indicating excellent overall performance. Sensitivity was 1.00, with no missed diagnoses. Specificity was 0.86, demonstrating strong discriminative ability. The PPV was 0.86, meaning 86% of positive predictions were correct. The NPV was 1.00, indicating perfect accuracy in identifying non-emergency cases (Figure 3). These performance metrics are summarized in Table 1.

**TABLE 1:** Accuracy of GPT in predicting emergency ultrasound indications.

Models	AUC	Sensitivity	Specificity	PPV	NPV
ChatGPT-4o	0.93	1.00	0.86	0.86	1.00

AUC, area under curve; PPV, positive predictive value; NPV, positive predictive value.

## 4 Discussion

This study evaluated the performance of the GPT-4O model in determining the indications for emergency ultrasound, and the results showed excellent performance, especially with a perfect sensitivity and NPV (both 1.00). This suggests that the GPT-4O model successfully identifies cases that meet the emergency ultrasound indications without missing any, while also accurately ruling out cases that do not meet the criteria. The model’s ability to reduce unnecessary ultrasound examinations, optimize resource allocation, and alleviate pressure on the healthcare system demonstrates its potential in clinical practice.

The confusion matrix and ROC analysis further supported the model’s effectiveness. The confusion matrix showed that the model correctly identified 93 non-emergency cases and 92 emergency cases, misclassifying only 15 non-emergency cases as emergencies, with no emergency cases misclassified as non-emergencies. The ROC curve, with an AUC of 0.93, further confirmed the model’s superior performance in binary classification tasks, suggesting that GPT-4O can be applied in high-volume emergency settings to provide quick and reliable decision support.

However, a deeper analysis of the 15 misclassified cases highlighted some challenges and areas for improvement. In some instances, the patients’ symptoms or chief complaints did not align with the guidelines for emergency ultrasound indications. For example, a pregnant woman with several days of diarrhea was mistakenly classified as meeting the emergency ultrasound criteria, though the ultrasound was intended for chronic monitoring, not acute assessment. This error suggests that the model’s understanding of symptoms like diarrhea may be too generalized, and its ability to precisely match clinical signs to guideline-based indications needs refinement (5).

Another challenge emerged when patients underwent multiple ultrasound exams for different body systems. For instance, a patient with acute cholecystitis required a gastrointestinal ultrasound for emergency evaluation, but also underwent a non-emergency urological ultrasound. The model, which did not differentiate between the various exam types, misclassified this case as meeting the emergency ultrasound criteria. This indicates that the model needs to improve its ability to evaluate individual ultrasound procedures within complex clinical scenarios (10).

These findings align with previous studies on AI applications in emergency medicine. For example, Arslan et al. (11) showed that ChatGPT and Copilot outperformed traditional nurse triage systems in high-volume emergency departments, with greater accuracy in identifying high-risk patients and stability across different populations. Similarly, Shekhar et al. (12) explored the triage potential of ChatGPT 4.0 in emergency services, finding that it aligned with experienced emergency nursing teams in most cases and demonstrated promising applications in resource allocation. This study expands the application of AI by verifying GPT-4O’s utility in assessing emergency ultrasound indications, demonstrating higher sensitivity and specificity than previous approaches (13).

Despite these promising results, the study has some limitations. First, it was designed as an exploratory validation based on a relatively small, single-center dataset, which limits the generalizability of the findings. This design may introduce selection bias and affect the model’s applicability to other healthcare settings. Additionally, although expert clinicians’ judgments were used as the reference standard, their subjective assessments may have influenced the evaluation, particularly in cases involving ambiguous symptoms or incomplete clinical information (14).

Future studies should involve multiple emergency centers to assess the robustness and applicability of GPT-4O across diverse patient populations. Additionally, exploring the integration of AI models into real-time clinical practice, where they can be compared dynamically with physician decisions, will be essential for evaluating the model’s real-world performance.

While the GPT-4O model demonstrated excellent sensitivity and overall performance, it is important to recognize the limitations inherent to large language models. These include susceptibility to hallucinations, dependence on prompt phrasing, and reduced performance in ambiguous or multi-system presentations. The model may also generalize overly from vague symptoms. Future efforts should focus on refining prompts and incorporating case complexity stratification to mitigate these risks.

In addition, integrating AI models into real-time emergency department workflows presents practical challenges. These include ensuring compatibility with electronic health record systems, gaining acceptance from clinicians, addressing liability concerns, and complying with regulatory standards. Thoughtful design, transparency, and clinical training will be essential for successful deployment.

In conclusion, this study demonstrates the significant potential of the GPT-4O model in determining emergency ultrasound indications. While further validation and optimization are needed, AI technology holds great promise for optimizing medical resource allocation and improving diagnostic efficiency in emergency medicine. Future studies should involve large-scale, multicenter, prospective cohorts to assess the robustness, generalizability, and real-world applicability of GPT-4O across diverse patient populations.

## Data Availability

The raw data supporting the conclusions of this article will be made available by the authors, without undue reservation.
